# Omega-3 Fatty Acids and Cardiovascular Disease: Are There Benefits?

**DOI:** 10.1007/s11936-016-0487-1

**Published:** 2016-10-17

**Authors:** Kate J. Bowen, William S. Harris, Penny M. Kris-Etherton

**Affiliations:** 1 Department of Nutritional Sciences, The Pennsylvania State University, 110 Chandlee Lab, University Park, PA 16802 USA; 2 Department of Internal Medicine, University of South Dakota Sanford School of Medicine and OmegaQuant Analytics, LLC, 5009 W. 12th St., Suite 8, Sioux Falls, SD 57106 USA

**Keywords:** Cardiovascular disease, Omega-3 fatty acids, EPA, DHA, Fish, Fish oil supplements

## Abstract

Early secondary prevention trials of fish and omega-3 polyunsaturated fatty acid (PUFA) capsules reported beneficial effects on cardiovascular disease (CVD) outcomes, including all-cause mortality and sudden cardiac death. These clinical findings, as well as observational and experimental data, demonstrated that omega-3 PUFAs reduced the risk of coronary outcomes and overall mortality and were the basis for recommendations made in the early 2000s to increase omega-3 PUFA intake. In the last 6 years, however, results from both primary and secondary prevention trials have generally failed to show a beneficial effect of omega-3 PUFA supplementation, bringing current recommendations into question. Several possible reasons for these null findings have been proposed, including short treatment periods, relatively low doses of omega-3 PUFAs, small sample sizes, higher background omega-3 intakes, and the concurrent use of modern pharmacotherapy for CVD prevention. At least one of these caveats is being assessed in major clinical trials, with two omega-3 PUFA pharmacological agents being tested at doses of 4 g/day (instead of the more common <1 g/day). These null findings, however, do not necessarily mean that omega-3 PUFAs “are ineffective” in general, only that they were not effective in the context in which they were tested. Accordingly, higher intakes of omega-3 PUFAs, either from fatty fish or from supplements, if continued for decades (as the epidemiological data support) are likely to contribute towards lower risk for CVD. At this time, evidence supports the consumption of a healthy dietary pattern with at least two servings per week of fatty fish. Omega-3 PUFA supplementation is a reasonable alternative for those who do not consume fish, although fish is the preferred source of omega-3 PUFAs because it also provides additional nutrients, some of which are often under-consumed.

## Introduction

The relationship between marine-derived omega-3 polyunsaturated fatty acids (PUFAs) and cardiovascular disease (CVD) originated from the early studies of Greenland’s Inuit population, an isolated cohort with a low incidence of ischemic heart disease and a dietary pattern comprised primarily of whales, seals, sea birds, and fish [1, 2]. Compared to a Danish cohort, this unique population had a less atherogenic lipid profile, lower platelet counts, higher omega-3 PUFAs in platelet membranes, and longer blood clotting times. It was hypothesized that the low incidence of CVD and reduced risk profile was a consequence of the antithrombotic effects of the distinct dietary pattern, high in the marine-derived omega-3 PUFAs eicosapentaenoic acid (EPA) and docosahexaenoic acid (DHA). These seminal findings were the basis for over four decades of observational and intervention studies conducted to evaluate the relationship between omega-3 PUFAs and CVD risk factors and events.

The preponderance of evidence from secondary prevention trials in the 1980s through the early 2000s demonstrated a cardioprotective effect of fish consumption and omega-3 PUFA supplementation [3–7]. Similarly, meta-analyses of observational studies reported an inverse relationship between fish consumption and fatal coronary heart disease (CHD) [8–10]. The growing evidence base led to seafood/fish and omega-3 PUFA intake recommendations from leading organizations, as well as a qualified health claim from the Food and Drug Administration (FDA) (issued in 2004): “Supportive but not conclusive research shows that consumption of EPA and DHA omega-3 fatty acids may reduce the risk of coronary heart disease. One serving of [Name of the food] provides [] gram of EPA and DHA omega-3 fatty acids [11].”

Data from recent prospective studies show an inverse relationship between biomarkers of marine-derived fatty acids, an objective measure of omega-3 PUFA consumption, and coronary outcomes [12, 13•]. A meta-analysis of 19 observational studies (*n* = 45,637) and fatty acid biomarkers (total plasma, phospholipids, cholesterol esters, and adipose tissue) using a continuous (per 1-SD increase) multivariable-adjusted analysis reported that EPA, docosapentaenoic acid (DPA), and DHA were associated with a 9–10 % lower risk of fatal CHD [RRs of 0.91 (95 % CI, 0.82–1.00) for EPA, 0.90 (95 % CI, 0.85–0.96) for DPA, and 0.90 (95 % CI, 0.84–0.96) for DHA] [13•]. In addition, the sum of EPA, DPA, and DHA was associated with an 11 % lower risk of fatal CHD (RR 0.89; 95 % CI, 0.84–0.95). A 2016 analysis of two prospective cohort studies, the Nurses’ Health Study (*n* = 83,349) and Health Professionals Follow-up Study (*n* = 42,884), found that intake of marine-derived omega-3 PUFAs was associated with lower total mortality when comparing extreme quintiles of intake (HR 0.96; 95 % CI, 0.93–1.00; *p* = 0.002 for trend) [14•].

In contrast to the collective observational evidence and early intervention trials, recent clinical trials have not reported beneficial effects of omega-3 PUFA supplementation on CVD outcomes [15–18, 19••]. Experts at the International Society for the Study of Fatty Acids and Lipids discussed experimental design issues that may be responsible for the null findings in recent investigations and must be considered in future clinical designs, such as an overshadowing effect of current CVD treatment on omega-3 PUFA benefits, high background intakes, small sample sizes, short treatment duration, insufficient dosage, increase in omega-6 PUFA intake, and failure to measure baseline omega-3 status [20•]. In 2016, the Agency for Healthcare Research and Quality published a systematic review of 61 randomized clinical trials (RCTs) and 37 longitudinal observational studies of omega-3 PUFAs and CVD risk factors or outcomes [21••]. The authors concluded there is high strength of evidence that increased marine oil intake improves high-density lipoprotein cholesterol (HDL-C) (0.9 mg/dL; 95 % CI, 0.2, 1.6), triglycerides (−24 mg/dL; 95 % CI, −31, −18), and the total cholesterol to HDL-C ratio (−0.17; 95 % CI, −0.26, −0.09), but does not affect risk of major adverse cardiovascular events, all-cause mortality, sudden cardiac death, coronary revascularization, or blood pressure. Notably, there is also high strength of evidence of increased intake and increased low-density lipoprotein cholesterol (LDL-C) (2.0 mg/dL; 95 % CI, 0.4, 3.6).

The divergent findings from the recent clinical trials (versus the earlier trials) have ignited debate and confusion about the relationship between omega-3 PUFAs and CVD outcomes. This paper summarizes the scientific evidence from key early RCTs that were the impetus for subsequent trials, reviews RCTs published in the last 6 years, and presents potential explanations for the discrepant results. A fatty acid primer, dietary sources of marine-derived omega-3 fatty acids, intake recommendations, and ongoing trials are discussed.

## Omega-3 fatty acid structure and physiological effects

- Structurally, fatty acids consist of a hydrocarbon chain with a carboxyl group (C(O)OH) and a methyl group (CH_3_) at opposing ends [22]. The absence of a double bond within the hydrocarbon chain denotes a saturated fatty acid, while the presence of one or more double bonds is characteristic of an unsaturated fatty acid. There are two types of unsaturated fatty acids, monounsaturated fatty acids containing one double bond and PUFAs containing two or more double bonds. Common convention describes unsaturated fatty acids based on the position of the first double bond relative to the methyl terminus carbon (“ω” or “n”). There are two subclasses of PUFAs, omega-3 and omega-6 fatty acids. Omega-3 fatty acids contain the first double bond between the third and fourth carbons from the methyl terminus and are classified as long chain fatty acids due to the carbon chain length.
- Omega-3 fatty acids of significance in human physiology and nutrition are α-linolenic acid (ALA; 18:3), EPA (20:5), and DHA (22:6) [22]. ALA is an essential fatty acid; it cannot be synthesized in humans and, thus, must be obtained through plant-based sources such as flaxseeds, walnuts, and canola oil. EPA and DHA are considered non-essential, as they can be synthesized from ALA via a series of desaturation and chain elongation steps. However, this pathway is inefficient, with human isotope studies reporting <0.1–7.9 % of ALA converted to EPA and <0.1–3.8 % of ALA converted to DHA [23]. Therefore, intake of marine-derived EPA and DHA is crucial for beneficial health effects.
- Mozaffarian and Wu reviewed the physiological effects of seafood-derived omega-3 PUFAs relative to cardiovascular health [24]. Omega-3 PUFAs have been shown in clinical settings to reduce triglycerides, blood pressure, and resting heart rate and improve endothelial dysfunction. Growing evidence suggests that omega-3 PUFAs may also reduce systemic vascular resistance and arrhythmias and improve myocardial efficiency and left ventricular diastolic filling. Antithrombotic properties have been suggested; however, the effect may only be apparent at very high doses (e.g., 15 g/day). In addition, omega-3 PUFA metabolites have anti-inflammatory effects, but the clinical relevance at dietary doses requires further investigation.

## Dietary sources and recommend intakes of EPA and DHA

- Fatty fish and seafood are the principal dietary sources of EPA and DHA (Table 1). Both wild and farm-raised varieties provide EPA and DHA, and some evidence suggests the latter may contain greater concentrations [27••]. In addition to their favorable fatty acid profiles, oily fish and seafood are nutrient dense dietary choices that are high in protein, low in saturated fat, and contain micronutrients (many of which are under-consumed), such as vitamin D, vitamin B12, selenium, potassium, and magnesium [28•] (Table 1). Higher trophic fatty fish (e.g., tilefish, king mackerel, shark, and swordfish) may be contaminated with methyl mercury or organic pollutants and should be avoided by vulnerable populations, particularly pregnant or breastfeeding women, those who may become pregnant, and children [26, 27••, 29••].

**Table 1:** Select macro- and micronutrient content per 100 g of seafood/fish

Dietary Source^a^	Variety^b^	EPA (g)	DHA (g)	EPA+DHA (g)	Protein (g)	VD (IU)	VB12 (μg)	Se (μg)	K (mg)	Mg (mg)
Salmon	Atlantic, farmed	0.69	1.457	2.147	22.1	526	2.8	41.4	384	30
	Atlantic, wild	0.411	1.429	1.84	25.44	–	3.05	46.8	628	37
	Chinook	1.01	0.727	1.737	25.72	–	2.87	46.8	505	122
	Coho, farmed	0.408	0.871	1.279	24.3	–	3.17	14.1	460	34
	Coho, wild	0.401	0.658	1.059	23.45	451	5	38	434	33
	Sockeye	0.299	0.56	0.859	26.48	670	4.47	35.5	436	36
	Chum	0.299	0.505	0.804	25.82	–	3.46	46.8	550	28
	Pink	0.218	0.399	0.617	24.58	522	4.73	37.6	439	32
Herring	Pacific	1.242	0.883	2.125	21.01	–	9.62	46.8	542	41
	Atlantic	0.909	1.105	2.014	23.03	214	13.14	15040	46.8	41
Mackerel	Pacific and Jack	0.653	1.195	1.848	25.73	457	4.23	46.8	521	36
	Spanish	0.294	0.952	1.246	23.59	–	7	40.6	554	38
	Atlantic	0.504	0.699	1.203	23.85	–	19	51.6	401	97
	King^c^	0.174	0.227	0.401	26	–	18	46.8	558	41
Sablefish		0.867	0.92	1.787	17.19	–	1.44	46.8	459	71
Tuna	Bluefin	0.363	1.141	1.504	29.91	–	10.88	46.8	323	64
	White, canned^d^	0.233	0.629	0.862	23.62	–	1.17	65.7	237	33
	Skipjack	0.091	0.237	0.328	28.21	–	2.19	46.8	522	44
	Light, canned^d^	0.047	0.223	0.27	25.51	–	2.99	80.4	237	27
	Yellowfin	0.015	0.105	0.12	29.15	82	2.35	108.2	527	42
Anchovy	European^e^	0.538	0.911	1.449	20.35	–	0.62	36.5	383	41
Halibut	Greenland	0.674	0.504	1.178	18.42	–	0.96	46.8	344	33
	Atlantic and Pacific	0.08	0.155	0.235	22.54	231	1.27	55.4	528	28
Bluefish		0.323	0.665	0.988	25.69	–	6.22	46.8	477	42
Sardine	Atlantic^f^	0.473	0.509	0.982	24.62	193	8.94	52.7	397	39
Bass	Striped	0.217	0.75	0.967	22.73	–	4.41	46.8	328	51
	Freshwater	0.305	0.458	0.763	24.18	–	2.31	16.2	456	38
	Sea	0.206	0.556	0.762	23.63	–	0.3	46.8	328	53
Trout	Mixed	0.259	0.677	0.936	26.63	–	7.49	16.2	463	28
	Rainbow	0.259	0.616	0.875	23.8	759	4.11	28.1	450	30
Tilefish^c^		0.172	0.733	0.905	24.49	–	2.5	51.5	512	33
Swordfish^c^		0.127	0.772	0.899	23.45	666	1.62	68.5	499	35
Shark^c,e^		0.316	0.527	0.843	20.98	24	1.49	36.5	160	49
Mussels	Blue^g^	0.276	0.506	0.782	23.8	–	24	89.6	268	37
Oyster	Pacific^e^	0.438	0.25	0.688	9.45	–	16	77	168	22
	Eastern, farmed^e^	0.188	0.203	0.391	5.22	–	16.2	63.7	124	33
	Eastern, wild^e^	0.177	0.136	0.313	5.71	1	8.75	19.7	156	18
Pollock	Atlantic	0.091	0.451	0.542	24.92	–	3.68	46.8	456	86
	Alaska^h^	0.086	0.423	0.509	23.48	51	3.66	44.1	430	81
Lobster	Spiny^g^	0.341	0.139	0.48	26.41	–	4.04	59.2	208	51
	Northern^g^	0.117	0.078	0.195	19	1	1.43	73.1	230	43
Queen	Queen^g^	0.332	0.145	0.477	23.72	–	10.38	44.4	200	63
	King^g^	0.295	0.118	0.413	19.35	–	11.5	40	262	63
	Dungess^g^	0.281	0.113	0.394	22.32	–	10.38	47.6	408	58
	Blue^g^	0.101	0.067	0.168	17.88	0	3.33	42.9	259	36
Snapper		0.048	0.273	0.321	26.3	–	3.5	49	522	37
Flatfish	Flounder and Sole	0.168	0.132	0.3	15.24	139	1.31	32.6	197	22
Clam^g^		0.138	0.146	0.284	25.55	–	98.89	64	628	18
Shrimp^g,h^		0.135	0.141	0.276	22.78	4	1.66	49.5	170	37
Scallop	Bay and Sea^g^	0.072	0.104	0.176	20.54	2	2.15	21.7	314	37

*EPA* eicosapentaenoic acid, *DHA* docosahexaenoic acid, *VD* vitamin D, *VB12* vitamin B12 (cobalamin), *Se* selenium, *K* potassium, *Mg* magnesium, − not available

^a^Data from the U.S. Department of Agriculture National Nutrient Database for Standard Reference, Release 28 (Software v.2.6.1) [25]

^b^All varieties cooked with dry heat unless otherwise specified

^c^Species with the highest levels of mercury [26]

^d^Canned in water, without salt, drained solids

^e^Raw

^f^Canned in oil, drained solids with bone

^g^Cooked with moist heat

^h^May have been previously frozen
- Krill, cod liver, and algal oils are non-traditional marine sources of long chain omega-3 fatty acids that have garnered attention as an alternative source of EPA and DHA. Future research is needed, however, to assess the health benefits of these oils.
- EPA and DHA are also available in omega-3 fortified foods, including breads, pastas, cereals, dairy products, eggs, meats, juices, salad dressings, spreads, and oils [30]. This is a result of fish oils (e.g., menhaden, salmon, tuna, anchovy) and algal oils directly added to foods, or animals given feed containing EPA and/or DHA to enrich their tissues. Consumption of omega-3 fortified foods is a potential option to increase EPA and DHA intake in vegans, vegetarians, or individuals who dislike fish/seafood.
- Leading dietary and cardiovascular health organizations issued intake recommendations for seafood/fish and marine-derived long chain fatty acids (Table 2). Notably, the FDA approved up to 3000 mg/day of EPA plus DHA from food and supplements as safe for the general population [36].

**Table 2:** Recommended intakes of seafood/fish and marine-derived long chain fatty acids

Organization	Population	Recommendation
Academy of Nutrition and Dietetics [31•]	Healthy adults	Two or more servings of fatty fish per week, providing at least 500 mg of EPA and DHA per day
American Heart Association [32–34]	All adults	Fish (particularly fatty) at least twice a week
	Patients with documented coronary heart disease	∼1 g of EPA and DHA (combined) per day^a^
	Patients with hypertriglyceridemia	2–4 g of EPA plus DHA per day provided as capsules under a physician’s care
	Women with hypercholesterolemia and/or hypertriglyceridemia	Omega-3 fatty acids in the form of fish or capsule (e.g., EPA 1800 mg/day) may be considered
U.S. Department of Agriculture, U.S. Department of Health and Human Services [29••]	General population	8 oz. per week of a variety of seafood, providing an average of 250 mg per day of EPA and DHA
	Pregnant or breastfeeding women	At least 8 and up to 12 oz. of a variety of seafood per week^b^
National Lipid Association [35•]	Adults	≥2 servings (3.5–4 oz.) of fish/seafood (preferably oily) per week^c^

*EPA* eicosapentaenoic acid, *DHA* docosahexaenoic acid

^a^Preferably from the consumption of oily fish; omega-3 fatty acid capsules can be considered in consultation with a physician

^b^Choose sources high in DHA and low in methyl mercury to optimize infant health outcomes

^c^Should not be prepared using deep-frying methods

## Timeline of early primary and secondary prevention trials

### 1980s

- The Diet and Reinfarction Trial (DART), published in 1989, was the first RCT to assess secondary prevention of myocardial infarction (MI) with dietary advice in 2,033 men recovered from previous MI [3]. The intervention group advised to consume ≥2 servings (200–400 g) of fatty fish per week had a 29 % reduction in risk of all-cause mortality (RR 0.71; 95 % CI, 0.54–0.93; *p* < 0.05) at 2 years versus the no advice control. A subset of participants who could not tolerate fish were given fish oil capsules (Maxepa 3 g/day); subgroup analyses indicated the effect of capsules was similar to the fatty fish treatment group [37].

### 1990s

- The Gruppo Italiano per lo Studio della Sopravvivenza nell’Infarto micardico Prevenzione (GISSI-Prevention) secondary prevention study included 11,323 participants (14.7 % female) with previous MI (≤3 months) and compared one fish oil capsule per day (850–882 mg EPA plus DHA as ethyl esters) in combination with the current standard of care versus standard of care alone for 3.5 years [4, 5]. Time course analyses revealed early effects of combination therapy; at 3 months, the intervention group had a 41 % reduction in risk of all-cause mortality (RR 0.59; 95 % CI, 0.36–0.97; *p* = 0.037) and, at 4 months, a 53 % reduction in risk of sudden cardiac death (RR 0.47; 95 % CI, 0.22–0.99; *p* = 0.048) [5]. At study completion, the reductions in risk of total death (RR 0.79; 95 % CI, 0.66–0.93; *p* = 0.006) and sudden death (RR 0.55; 95 % CI, 0.39–0.77; *p* = 0.0006) remained significant. The authors concluded the lowered risk of all-cause mortality was driven primarily by the reduction in sudden cardiac death and hypothesized an antiarrhythmic effect of EPA and DHA.

### 2000s

- The Diet And Reinfarction Trial 2 (DART-2) was a secondary prevention trial conducted in men with stable angina, rather than previous MI as was done in DART-1 [38]. Participants (*n* = 3,114) advised to eat two portions of oily fish per week (or 3 g/day of fish oil capsules) had a higher risk of cardiac death (HR 1.26; 95 % CI, 1.00–1.58; *p* = 0.047) and sudden cardiac death (HR 1.54; 95 % CI, 1.06–2.23; *p* = 0.025) at 3–9 years of follow-up versus the control of non-specific, sensible eating advice. Subgroup analyses revealed these results were driven by the fish oil capsule group (*n* = 462), not the dietary fish advice group (*n* = 1,106), as evident by a higher risk of cardiac death (HR 1.45; 95 % CI, 1.05–1.99; *p* = 0.024) and sudden cardiac death (HR 1.84; 95 % CI, 1.11–3.05; *p* = 0.018) in capsule compared to controls. However, the quality of DART-2 and its experimental design limitations, such as lack of blinding, study interruption at 1 year, assessment of compliance from a small subset, and similarities in background fish consumption between treatment and control, have been criticized [39].
- Investigators in Japan evaluated the effects of statin plus 1800 mg EPA daily versus statin alone in the Japan EPA Lipid Intervention Study (JELIS) [6]. This combined primary (*n* = 14,981) and secondary intervention (*n* = 3,664) trial followed participants (69 % female) a mean 4.6 years and reported the statin with EPA cohort had a 19 % reduction in major coronary events (HR 0.81; 95 % CI, 0.69–0.95; *p* = 0.011). Subgroup analyses revealed those with a history of coronary artery disease (defined as MI > 6 months, coronary interventions, or stable angina pectoris) had a 19 % reduction in major coronary events (HR 0.81; 95 % CI, 0.66–1.00; *p* = 0.048), while those without a history of coronary artery disease did not have a significant reduction in major coronary events (HR 0.82; 95 % CI, 0.63–1.06; *p* = 0.132). The null findings in the primary prevention group were attributed to a lack of power to detect significance. Notably, the risk of sudden cardiac death did not differ between treatment and control in the combined (*p* = 0.854) and subgroup analyses (primary *p* = 0.736, secondary *p* = 0.967).
- GISSI-Heart Failure (GISSI-HF) was the first large, randomized, double-blind, placebo-controlled trial conducted to assess the efficacy of omega-3 fatty acids (850–882 mg EPA plus DHA as ethyl esters) versus placebo in men and women with chronic heart failure (*n* = 6,975) [7]. At a median 3.9 years of follow-up, patients in the omega-3 group had a 9 % reduction in all-cause mortality (HR 0.91; 95.5 % CI, 0.833–0.998; *p* = 0.041) and an 8 % reduction in cardiovascular hospital admissions (HR 0.92; 99 % CI, 0.849–0.999; *p* = 0.009) compared to placebo. This moderate reduction in events with EPA plus DHA was observed in the context of standard of care practices in the early 2000s.

## Recent RCTs (2010–2016)

### Alpha Omega (2010)

- The Alpha Omega trial was a double-blind, placebo-controlled, randomized, secondary prevention study in patients with a MI in the past 10 years (median 3.7 years) [15]. In contrast with previous study interventions, this trial provided low dose omega-3 fatty acids or placebo in margarine for daily consumption for 40 months.
- Patients in the EPA plus DHA arms consumed 18.8 g/day of margarine containing 226 mg EPA and 150 mg DHA. The study sample (*n* = 4,837) was primarily older men (78 % men; mean age = 69 years) with almost all participants receiving therapies at baseline (antithrombotic (97.5 %), antihypertensive (89.7 %), and lipid modifying (86 %)). After a median follow-up of 40.8 months, the EPA plus DHA containing margarines did not reduce the rate of major cardiovascular events (composite of fatal and non-fatal cardiovascular events and cardiac interventions) compared to non-EPA and DHA margarines (336 (14 %) vs. 335 (13.8 %)) (HR 1.01; 95 % CI, 0.87–1.17; *p* = 0.93). All secondary a priori outcomes were null.
- Both GISSI-Prevention [4, 5] and Alpha Omega were secondary prevention trials of omega-3 fatty acids conducted for approximately 3.5 years in primarily male patients with previous MI. There are notable differences between the trials; GISSI-Prevention provided roughly double the dose of EPA and DHA, had a significantly larger sample size, delivered the intervention in oral supplement form, included higher risk patients with recent MI (median 16 days), had a younger sample (mean age at baseline 59.3 years), and followed standard of care practices from the 1990s (e.g., 4.7 % of patients on cholesterol-lowering therapies at baseline and 45.5 % at end of study) [4, 5]. JELIS was comparable to Alpha Omega in medication use (100 % of JELIS participants prescribed statins) and sample size in the secondary prevention cohort; however, the differing outcomes may be attributed to the eight times higher dose of EPA in JELIS provided to a primarily female, younger cohort (mean age at baseline 61 years) for a longer duration [6]. In addition, the Alpha Omega comparison group for statistical analyses was not a true placebo, but rather a composite of placebo or ALA-containing margarines.

### OMEGA (2010)

- The purpose of OMEGA was to evaluate the addition of omega-3 PUFA supplementation to current guideline-adjusted therapy on the prognosis of recent MI survivors (3–14 days) [16]. This randomized, placebo-controlled, double-blind trial compared 1 g/day of omega-3 capsules (460 mg EPA and 380 mg DHA as ethyl esters-90) versus 1 g/day of olive oil control in the context of current treatment.
- The investigators enrolled 3,851 patients (74.4 % male; median age 64 years). At 1 year of follow-up, event rates for all outcomes did not differ between the intervention and control groups, including sudden cardiac death (*p* = 0.84), all-cause mortality (*p* = 0.18), major adverse cerebrovascular or cardiovascular events (*p* = 0.1), and revascularization (*p* = 0.34).
- The lack of significant findings was likely the result of an underpowered study; the authors acknowledged they did not reach 80 % statistical power and, after revised calculations, would require a sample size of 20,000 to detect a significant effect. This was due to an over estimation of the effect of omega-3 fatty acids, as well as the lower than expected event rates (e.g., total mortality and sudden cardiac death), indicative of improved clinical care. In addition, both groups increased their fish consumption from baseline to study completion, potentially causing comparable total omega-3 PUFA intakes between groups. Moreover, the study duration of a 1-year supplementation period and follow-up may not have been sufficient.

### SU.FOL.OM3 (2010)

- Supplémentation en Folates et Omega-3 (SU.FOL.OM3) was a double-blind, randomized, placebo-controlled, 2 × 2 factorial trial of B vitamin supplementation (560 μg 5-methyltetrahydrofolate, B6, and B12) or placebo and omega-3 fatty acids (600 mg EPA and DHA) or placebo in patients with an acute coronary (MI or acute coronary syndrome) or cerebral ischemic event in the past 12 months [17].
- At baseline, the median time from event to randomization for all study participants (*n* = 2,501) was 101 days. During a median follow-up of 4.7 years, omega-3 fatty acids did not significantly reduce major vascular events (composite of non-fatal MI, stroke, or death from CVD) (81 (6.5 %) vs. 76 (6.1 %)) (HR 1.08; 95 % CI, 0.79–1.47; *p* = 0.64). All secondary outcomes were not significantly different between omega-3 supplementation and placebo.
- SU.FOL.OM3 was underpowered due to a 15 % lower than anticipated event rate; standard of care pharmacotherapy practices were prevalent (e.g., 93 % using Aspirin/anti-platelet therapy, 85 % using lipid lowering agents) and likely contributed to the low event rate. Additionally, the interval from initial event to omega-3 supplementation may have been too lengthy; the beneficial effects of omega-3 fatty acids may be more discernable in the acute phase.

### ORIGIN (2012)

- The Outcome Reduction with an Initial Glargine Intervention (ORIGIN) trial was the first large study to investigate the efficacy of long-term omega-3 fatty acid supplementation on cardiovascular events in individuals with CVD plus impaired glucose tolerance, impaired fasting glucose, or diabetes [18]. This trial was a double-blind, randomized, placebo-controlled, 2 × 2 factorial design of 1 g/day of fish oil (465 mg EPA and 375 mg DHA as ethyl esters) versus 1 g/day olive oil placebo and insulin glargine versus standard care.
- The investigators followed 12,536 patients for a median of 6.2 years and reported the primary outcome of death from cardiovascular causes was not significantly decreased in the omega-3 group compared to placebo (574 (9.1 %) vs. 581(9.3 %)) (HR 0.98; 95 % CI, 0.87–1.10; *p* = 0.72). In addition, all other study outcomes, including rates of major vascular events (MI, stroke, death from cardiovascular causes), all-cause mortality, and death from arrhythmia, were not significantly reduced in the omega-3 group versus placebo.
- The ORIGIN findings are in contrast with the GISSI-Prevention and GISSI-HF results, despite comparable supplement dose and fatty acid form across studies. The differing populations for secondary prevention may explain this discrepancy: GISSI-Prevention patients had a MI in the past 3 months (median 16 days) [4, 5], GISSI-HF patients had chronic heart failure [7], and ORIGIN patients had dysglycemia and evidence of CVD with more inclusive criteria. Another potential explanation for the lack of significant findings is the use of concomitant therapies among the ORIGIN population, thereby preventing a detectable effect of low dose omega-3 supplementation. Higher doses may be necessary with advanced medical care.

### Risk and Prevention (2013)

- The Risk and Prevention study was a randomized, double-blind, placebo-controlled trial that assessed the efficacy of 1 g/day of omega-3 fatty acids (850–882 mg EPA and DHA as ethyl esters) versus olive oil placebo in an Italian cohort of patients at high CVD risk without previous MI [19••, 40]. Both arms included standard of care treatment and preventive strategies, such as lifestyle interventions and pharmacological therapies. The primary endpoint was originally the cumulative rate of death, non-fatal MI, and non-fatal stroke; however, following intermediate blinded analyses that revealed low event rates, the primary endpoint was changed to the composite of death or hospitalization from cardiovascular cause [19••].
- The investigators enrolled 12,513 patients with a median follow-up of 5 years. Intent to treat analyses (*n* = 12,505) showed the primary endpoint was not significantly reduced in the omega-3 group compared to placebo (733 (11.7 %) vs. 745 (11.9 %)) (HR 0.98; 95 % CI, 0.88–1.08; *p* = 0.64) and the secondary endpoints were null. Subgroup analyses revealed significantly fewer heart failure hospitalizations in the omega-3 group (96 (1.5 %) vs. 142 (2.3 %); *p* = 0.002) and women in the omega-3 group had lower rates of the primary endpoint (187 (8 %) vs. 237 (9.6 %)) (HR 0.82; 95 % CI, 0.67–0.99; *p* = 0.04).
- The authors acknowledged the lower than expected rates of hard cardiovascular endpoints as a trial limitation. This may be a consequence of inclusion of an Italian cohort following a Mediterranean dietary pattern and/or effective preventive standard of care practices. Similar to the OMEGA [16] and ORIGIN trials [18], the Risk and Prevention study used olive oil as the placebo. In the PREDIMED (Prevención con Dieta Mediterránea) trial, a Mediterranean diet supplemented with extra-virgin olive oil (50 g/day) reduced the incidence of major cardiovascular events (composite of myocardial infarction, stroke, or death from cardiovascular cause) at 5 years of follow-up [41]. The reduction in major cardiovascular events may have been a result of the Mediterranean dietary pattern, the olive oil, or a combined effect. If olive oil alone is responsible for the observed benefits, it may not be an appropriate control in omega-3 fatty acid trials; however, low dose olive oil (i.e., 1 g/day) is unlikely to affect cardiovascular outcomes.

## Ongoing clinical trials

- Four ongoing studies will provide further evidence of the efficacy of omega-3 supplementation in both generally healthy, low-risk groups, as well as high-risk populations. Below are summaries of the study designs, target populations, primary outcomes, and expected completion dates. The results of these CVD prevention trials are expected to be available in 2018–2019.
- A Study of Cardiovascular Events in Diabetes (ASCEND) is a randomized, placebo-controlled, double-blind, 2 × 2 factorial trial of 100 mg/day aspirin or placebo and 1 g/day omega-3 fatty acids (400 mg EPA plus 300 DHA as ethyl esters) or olive oil placebo in 15,480 patients ≥40 years with diabetes (type 1 or 2) and without a history of vascular disease [42, 43]. Participants in this primary prevention trial will be followed on average 5–7 years for serious vascular events (defined as non-fatal MI, non-fatal stroke or transient ischemic attack, or vascular death excluding cerebral hemorrhage). The study began in 2004 and the estimated completion date is September 2017 (ClinicalTrials.gov Identifier NCT00135226).
- The Vitamin D and Omega-3 Trial (VITAL) is the first trial of omega-3 supplements for the primary prevention of cancer and CVD in the general population without cancer or CVD at baseline [44, 45]. VITAL is a randomized, double-blind, placebo-controlled, 2 × 2 factorial study of vitamin D_3_ (2000 IU/d) versus placebo and 1 g/day Omacor fish oil supplement (465 mg EPA plus 375 mg DHA) versus placebo. The primary outcome is risk of total cancer and major CVD events (MI, stroke, cardiovascular mortality). Enrollment and randomization began in November 2011 and was completed in March 2014, with a total sample size of 25,875 males (≥50 years) and females (≥55 years) and an over sampling of blacks [46]. The mean follow-up is a predicted 5 years and the estimated completion date is December 2017 (ClinicalTrials.gov Identifier NCT01169259).
- The Reduction of Cardiovascular Events with EPA-Intervention Trial (REDUCE-IT) is a randomized, parallel arm, double-blind trial to compare 4 g/day of Vascepa (EPA ethyl ester) with statin therapy versus statin therapy alone in reducing cardiovascular events (composite of cardiovascular death, MI, stroke, coronary revascularization, and hospitalization for angina) [47, 48]. This is the first study to assess high dose EPA plus statin in a Western population with hypertriglyceridemia despite statin use. Eligibility criteria include hypertriglyceridemia, established CVD or high risk for CVD, and aged ≥45 years. A predicted 8,000 participants will be followed for 4–6 years. The trial commenced in 2011 and the estimated completion is 2017, with results available in 2018 (ClinicalTrials.gov Identifier NCT01492361).
- STRENGTH (Statin Residual Risk Reduction with Epanova in High Risk Patients with Hypertriglyceridemia) is a randomized, double-blind, parallel arm, placebo-controlled trial of 4 g/day Epanova (omega-3 carboxylic acids) plus statin versus corn oil placebo plus statin [49]. Participants (target *n* = 13,000) 18–99 years with optimal LDL-C levels, hypertriglyceridemia, low HDL-C, and high CVD risk will be followed 3–5 years for major atherosclerotic coronary events (composite of cardiovascular death, non-fatal MI or stroke, coronary revascularization, or angina hospitalization). Study enrollment began in 2014 and the anticipated study completion date is 2019 (ClinicalTrials.gov Identifier NCT02104817).

## Conclusions

Results from early trials of fish consumption and omega-3 PUFA supplementation, as well the collective observational data, demonstrate a cardioprotective effect of long chain omega-3 fatty acids. In contrast, primary and secondary prevention trials published in the last 6 years report null effects of supplementation and call in to question the efficacy of low dose omega-3 PUFAs in reducing cardiovascular events. Potential explanations for the discrepant results include underpowered studies with small samples and low event rates, participants with high background fish/seafood intakes, suboptimal EPA and DHA dosage, supplementation duration, age at study enrollment, length of follow-up, and concurrent standard of care for CVD treatment. Notably, supplementation of 1 g/day of EPA plus DHA is unlikely to affect CVD outcomes in the context of modern CVD treatment with multiple pharmacotherapies. Four ongoing trials address numerous design limitations, and their results will provide crucial insight into the effects of EPA and DHA supplementation on CVD outcomes.

Based on the available evidence, physicians should advise patients to consume a healthy dietary pattern that includes at least two servings of fatty fish per week. This is an important adjunct to the standard of care for CVD treatment. For individuals who do not consume omega-3 rich fish, fish oil capsules as a source of EPA and DHA need not be discouraged, given their long safety record and the benefit to risk ratio; however, LDL-C levels should be monitored, especially in hypertriglyceridemic patients being treated with high doses of omega-3 fatty acids. Nevertheless, a food-based approach is the preferred option to deliver not only the marine-derived omega-3 PUFAs EPA and DHA, but also other beneficial nutrients.
